# High Proton Selectivity Sulfonated Polyimides Ion Exchange Membranes for Vanadium Flow Batteries

**DOI:** 10.3390/polym10121315

**Published:** 2018-11-27

**Authors:** Qi Chen, Liming Ding, Lihua Wang, Haijun Yang, Xinhai Yu

**Affiliations:** 1 Key Laboratory of High Performance Fibers & Products, Ministry of Education, Donghua University, Shanghai 201620, China; chenqitop01@126.com; 2 Key laboratory of Green Printing, Institute of Chemistry Chinese Academy of Sciences, Beijing 100190, China; 3120160545@bit.edu.cn (L.D.); yanghj@iccas.ac.cn (H.Y.)

**Keywords:** sulfonated polyimides, ion exchange membrane, proton selectivity, Vanadium flow batteries

## Abstract

High proton selectivity is the ultimate aim for the ion exchange membranes (IEMs). In this study, two kinds of sulfonated polyimides (SPI)—non-fluorinated and fluorine-containing polyimide—with about 40% sulfonation degree were synthesized by one-step high temperature polymerization. High proton selectivity IEMs were prepared and applied in vanadium flow batteries (VFB). The chemical structures, physicochemical properties and single cell performance of these membranes were characterized. The results indicate that high molecular weight of SPIs can guarantee the simultaneous achievement of good mechanical and oxidative stability for IEMs. Meanwhile, the proton selectivity of SPI membrane is five times higher than that of Nafion115 membranes due to the introduction of fluorocarbon groups. Consequently, the single cell assembled with SPI membranes exhibits excellent energy efficiency up to 84.8% at a current density of 100 mA·cm^−2^, which is 4.6% higher than Nafion115. In addition, the capacity retention is great after 500 charge–discharge cycles. All results demonstrate that fluorinated SPI ion exchange membrane has a bright prospect in new energy field.

## 1. Introduction

New energy sources, such as solar, wind, geothermal, and hydro energy have gained wide attention during the past decades because they are renewable and eco-friendly [1,2,3]. However, the discontinuous, unstable, and uncontrollable output of those energies limits their practical application to a large extent. Vanadium flow battery (VFB) is a kind of large-scale energy storage system which features with high energy efficiency, long cycle life, less pollution to the environment, controllable capacity, and low requirement for geography [4,5]. Therefore, VFBs have attracted continuous attention since they were first put forward by Skyllas-Kazacos in 1985 [6,7,8,9]. As one of the key components of VFB, ion exchange membranes (IEMs) can directly influence the lifespan of VFB. Therefore, high proton conductivity and good mechanical and chemical stability are desirable merits for IEMs to achieve high performance [5,10,11,12,13].

Nowadays, Nafion^®^ series membranes invented by DuPont are widespread used as IEMs for VFB. The advantages of Nafion^®^ series membranes include remarkable and high proton conductivity, chemical and mechanical stability, and so on. However, their shortcomings are also obvious, such as serious vanadium permeability and high cost [14,15]. The water channel formed by the hydrophobic main chain and hydrophilic side chain cause severe permeation of vanadium, and the perfluorinated backbone makes them expensive [14,16]. In order to overcome the shortcomings of Nafion membranes, researchers have made great efforts during the past few decades, such as modification of perfluoro sulfonic IEMs by blending [15], synthesis of non-fluorinated IEMs [17], preparation of porous membranes [18], and so forth [19]. Non-fluorinated IEMs have gained wide attention due to the fact that they are non-fluorinated polymers and their preparation cost is usually low. In previous studies, non-fluorinated sulfonated polysulfone (SPSF) [20,21], sulfonated polyimide (SPI) [22,23,24,25] and sulfonated poly (ether ether ketone) (SPEEK) [26,27,28,29] have been deeply investigated.

Polyimides are a class of polymers containing imide groups in the main chain, they not only have good mechanical property, excellent proton conductivity, and stable thermal stability [30,31], but they also demonstrate structural diversity and easy film-forming capability [32]. Therefore, SPI is a promising candidate to prepare IEMs for VFB application. Zhang et al. [33] synthesized a series of sulfonated polyimides (SPIs) membranes by 1,4,5,8-naphthalenetetracarboxylicdianhydride (NTDA), 2,2′-benzidinedisulfonic acid (BDSA), and 4,4′-diaminodiphenyl ether (ODA) with different sulfonation degree. The physicochemical properties of those membranes, such as water uptake, ion exchange capacity, proton conductivity, and permeability of vanadium ion were well characterized. Proton conductivity of SPI membranes increased from 0.012 to 0.051 S·cm^−1^ for the sulfonation degree ranged from 40% to 89%, and the permeability of vanadium ion was much lower than Nafion117. Therefore, they concluded that SPIs show potential value in application for vanadium redox flow batteries. This research group prepared three kinds of SPI membranes for VFB applications by changing the used diamines [32,34,35]. All membranes show excellent vanadium ion blocking, the permeability of vanadium ions were one or two orders of magnitude lower than Nafion117. The coulombic efficiency of SPI membranes could reach more than 97% at the current density of 30 mA·cm^−2^, and SPI(BAPP) which use 2,2-bis[4-(4-aminophenoxy)phenyl] propane as diamine monomer has the best VFB performance, the energy efficiency of SPI(BAPP) was 78% at the current density of 30 mA·cm^−2^, about 13% higher than that of Nafion117. They also synthesized a kind of branched sulfonated polyimide (bSPI) to prepare membranes for VFB application, and the degree of branching (DB) of membranes was investigated in detail. The results indicated that bSPI showed better thermal and mechanical properties than linear SPI; proton conductivity and vanadium ion permeability both increased with the development of DB. The overall performance of bSPI membrane with 8% of DB value (bSPI-8) was the best. The VRFB system assembled with bSPI-8 membrane showed higher coulombic and energy efficiency than Nafion117 at the current density ranging from 50 to 120 mA·cm^−2^. However, more studies have been dedicated to the SPIs’ blending modification. Zwitterionic polymer functionalized grapheme oxide (ZGO) [36], sulfonated molybdenum disulfide (*s*-MoS_2_) [37], ZrO_2_ [38], AlOOH [34], tungsten phosphoric acid (ATP) [39], and some other inorganic particles [40,41,42,43] have been introduced into SPI matrix to fabricate hybrid membranes for VFB due to the fact that the introduced inorganic particles could usually improve the proton conductivity as well as reduce the vanadium permeability.

In order to pursue the appropriate polymer materials that have good mechanical properties and high proton conductivity for VFB, herein, two kinds of SPI with almost the same degree of sulfonation were used as IEMs for VFB applications. Especially, the introducing of C–F bond in SPI chain would play an equilibrating action role between water uptake and vanadium ion permeability. The microstructure, physicochemical properties, and proton selectivity of the IEMs were systematically investigated. It is important that the single cell performance, open circuit voltage, and capacity retention were tested for 500 charge–discharge cycles to further verify the service life of SPI ion exchange membranes.

## 2. Experimental

### 2.1. Materials

1,4,5,8-naphthalenetetracarboxylic dianhydride (NTDA), 4,4′-oxydiphthalic anhydride (ODPA) and *m*-cresol were purchased from Sinopharm Chemical Reagent Beijing Co., Ltd. (Beijing, China). 2,2′-benzidinedisulfonic acid (BDSA) and 2-bis(4-(4-aminophenoxy)phenyl)hexafluropropane (HFBAPP) were supplied by Tokyo Kasei Co. (Tokyo, Japan). 4,4′-diaminodiphenyl ether (ODA) was purchased from J&K Scientific Ltd. (Beijing, China). Triethylamine (Et_3_N) and benzoic acid were provided by Xilong Chemical Co., Ltd. (Nanchang, China). Nafion115 (N115) membranes were supplied by DuPont Co. (Wilmington, NC, USA). ODPA and NTDA were dried under vacuum before use. BDSA and ODA were purified before use.

### 2.2. Synthesis of Polyimides

SPI was synthesized by a typical one-step high temperature polymerization method, a typical synthetic procedure mixes BDSA (3.4436 g, 0.010 mol) and triethylamine (2.4286 g, 0.024 mol) with *m*-cresol (110 mL) in four flasks under the protection of nitrogen. Then, ODA or HFBAPP (2.0024 g or 4.1052 g, 0.010 mol), benzoic acid (3.4194 g, 0.028 mol), and NDTA (5.3636 g, 0.020 mol) are added into the solution, stirred at 80 °C for 4 h, then heated up for 180 °C and reacted for 20 h. After reaction, the product is cooled to 80 °C, then the viscous solution is poured into excessive acetone slowly to get the polyimide precipitation. The precipitation is washed several times with acetone and then dried under vacuum at 100 °C for 12 h.

### 2.3. Preparation of SPI Membranes

The membranes were prepared by a typical solution casting method, SPI was added into DMAc and then stirred at 70 °C until it was completely dissolved. The homogeneous solution was cast on a clean glass pane and dried under an infrared lamp for 2 h, then dried at 100 °C in vacuum for 12 h. After that, the glass pane was soaked in deionized water and the membrane was peeled off. Then the membrane was soaked into 1M H_2_SO_4_ for 72 h and washed by deionized water for use. The membranes were recorded as SPI-ODA and SPI-HFBAPP, respectively.

### 2.4. Characterization of SPI

Fourier transform infrared spectroscopy (FT-IR, Thermo Scientific, Waltham, MA, USA) and nuclear magnetic resonance (^1^H-NMR, Bruker, Rheinstetten, Germany) were used to characterize the structure of SPI. The samples were mixed with KBr and pressed into pellet and FT-IR spectra in absorbance mode were recorded among the range of 500–4000 cm^−1^. The ^1^H-NMR spectra was recorded by using DMSO-d_6_ as a solvent.

Thermogravimetric (TG, Netzsch, Germany) was used to characterize the thermal stability of SPI; the products were evaluated from 30 °C to 900 °C in nitrogen at a heating rate of 20 K·min^−1^.

Chromatography (GPC, Milford, PA, USA) was performed to measure the molecular weight of the polymer, DMF was used as solvent.

### 2.5. Characterization of Membranes

The water uptake (WU) was measured by weighting and calculating according to Formula (1)

(1) $$\;\mathrm{WU}\;\left(\%\right)\;=\;\frac{W_{w}\;-\;W_{d}}{W_{d}}\;\times \;100\%$$

where $W_{d}$ is the dry weight of membranes and $W_{w}$ is the wet weight of membranes after dipping in deionized water for 12 h at environment temperature.

The area resistance (AR) was characterized by EIS (Zahner Zennium) [44], and the proton conductivity was calculated using Equation (2)

(2) $$\delta \;=\;\frac{L}{R}$$

where L and R denote the thickness and AR of the membrane, respectively.

The ion exchange capacity (IEC) of membranes was obtained by a titration method. The dried membranes were immersed in 1M NaCl for 24 h at room temperature and then the NaCl solutions were titrated by 0.01 M NaOH. The IEC was calculated by Equation (3)

(3) $$\;\mathrm{IEC}\;=\;\frac{V_{NaOH}\cdot C_{NaOH}}{W_{d}}$$

where $V_{NaOH}$ is the volume of NaOH and $C_{NaOH}$ is the concentration of NaOH, $W_{d}$ is the dried weight of membranes.

The vanadium permeability (*P*) was calculated using the Equation (4)

(4) $$V_{R}\frac{dC_{R}\left(t\right)}{dt}\;=\;A\frac{P}{L}\left[C_{L}\;-\;C_{R}\left(T\right)\right]\;$$

where $V_{R}$ is the volume of the right reservoir (160 mL) and $\frac{dC_{R}\left(t\right)}{dt}$ represents the vanadium concentration in the right reservoir as a function of time; A and L represent the area and thickness of the membranes, respectively. $C_{L}$ represents the vanadium concentration in the left reservoir. The detailed test procedure was referred to our published paper [28]. 

The single cell performance was tested using A LAND CT2001A (LANHE, Wuhan, China) and the battery size was the same as that mentioned in [28].

## 3. Results and Discussion

### 3.1. Structure Characterization of SPI

**Scheme 1:**
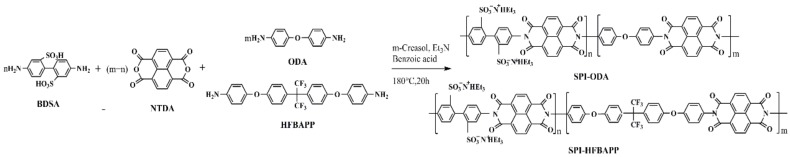
Synthesis of SPI.

**Figure 1:**
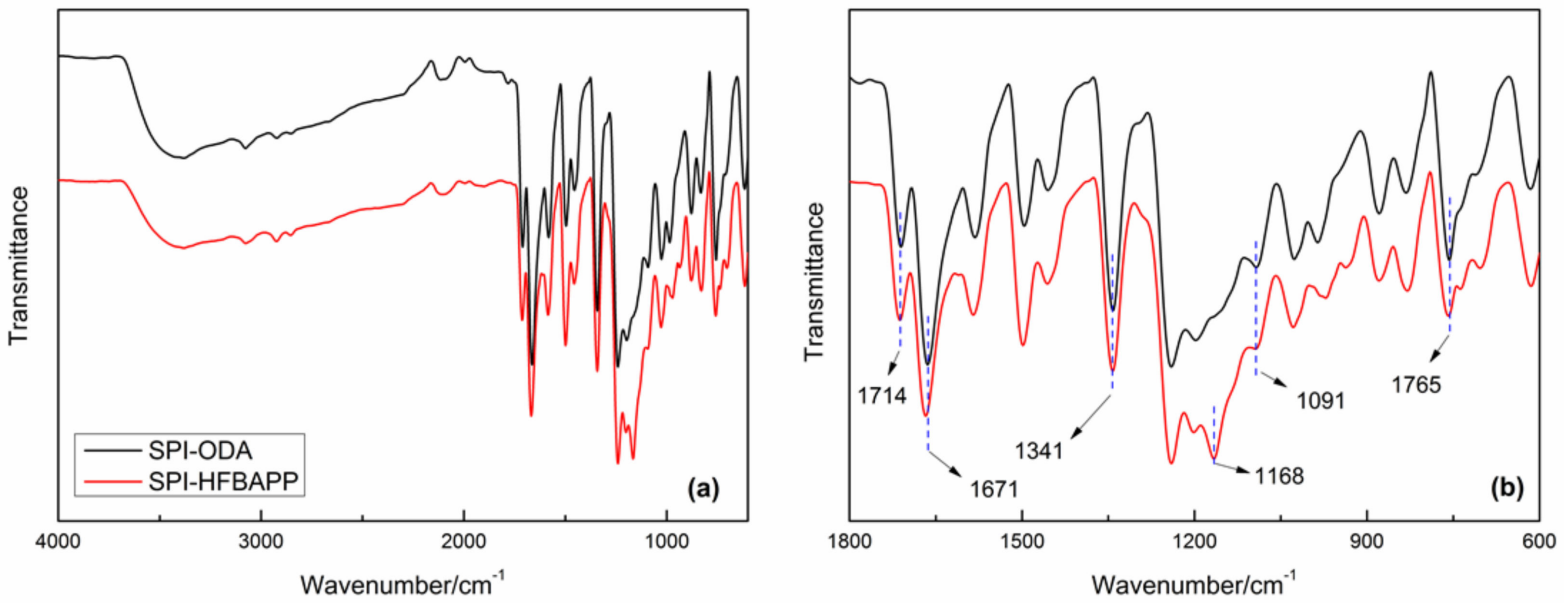
FT-IR of SPI-ODA and SPI-HFBAPP. (**a**) Wavenumber from 500 cm^−1^ to 4000 cm^−1^; (**b**) wavenumber from 600 cm^−1^ to 1800 cm^−1^.

SPI was prepared by a typical one-step high temperature polymerization method, and the corresponding chemical reaction equations were shown in Scheme 1. The chemical structure of the polymer was characterized by FT-IR and ^1^H-NMR. It can be seen from Figure 1, the absorption peak at 1714 cm^−1^ belongs to the asymmetric stretching of carbonyl group, while 1674 cm^−1^ belongs to the symmetric stretching of carbonyl group, 1341 cm^−1^ is the stretching vibration of C–N–C and 765 cm^−1^ is the out-of-plane blending vibration of imide ring. The absorption band around 1091cm^−1^ indicates the presenting of S=O. The above characteristics manifest the successful synthesis of SPI-ODA. The absorption peak at around 1168 cm^−1^ is the characteristic peak of C–F vibration, indicating the successful introduction of HFBAPP onto the SPI-HFBAPP chain. 

**Figure 2:**
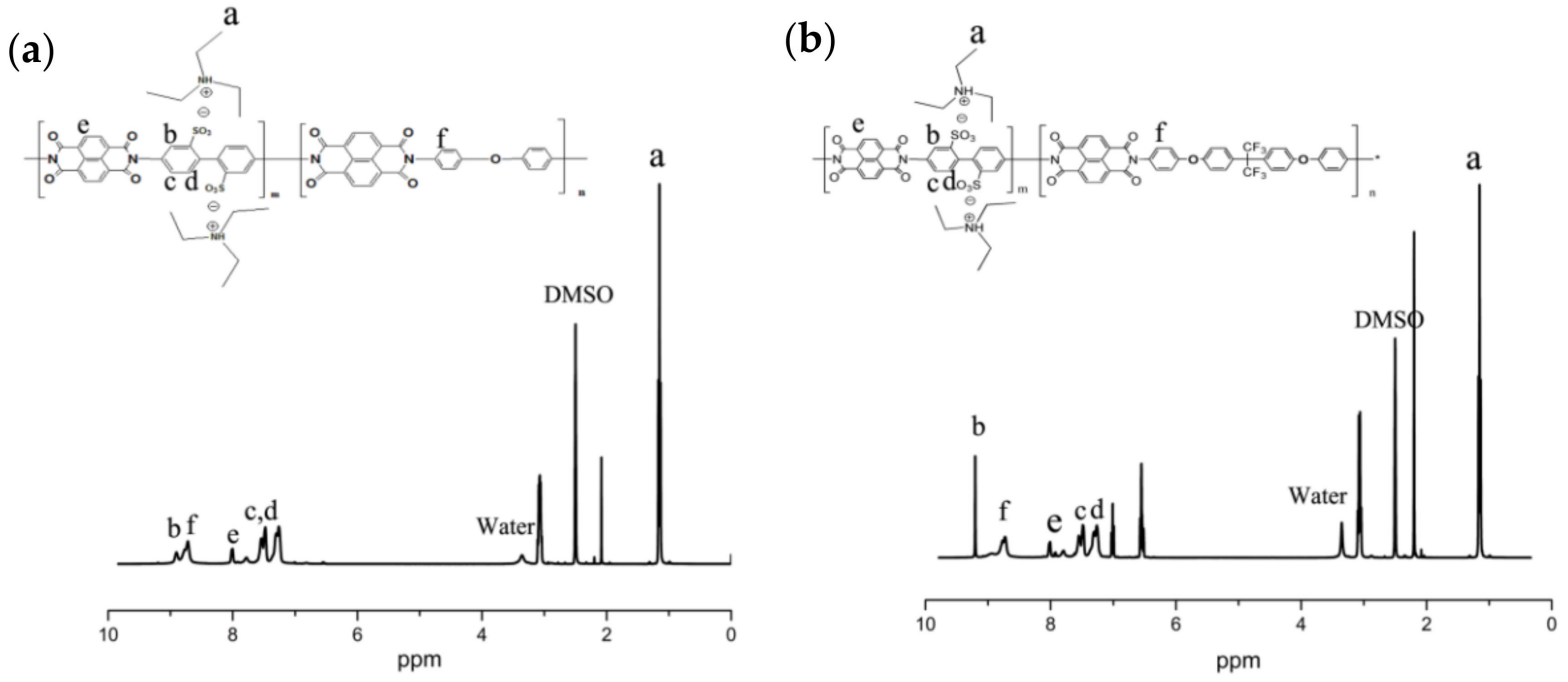
^1^H-NMR spectrum of SPI-ODA and SPI-HFBAPP. (**a**) SPI-ODA; (**b**)SPI-HFBAPP.

^1^H-NMR spectrums of SPI are shown in Figure 2. The spectroscopy peaks could be divided into two groups, the one at about 1.5 ppm is generated by Et_3_N, while the chemical shift between 9.3 ppm and 7.3 ppm come from aromatic ring. Three appeared peaks at 6.6, 7.0, and 9.26 ppm belong to m-cresol for SPI-HFBAPP [22], therefore, by integration, the degree of sulfonation (DS) of SPI-ODA and SPI-HFBAPP could be calculated accurately by the following Formulae (5) and (6). Although the molar ratio of BDSA to the total diamine in the reactants is up to 50%, the actual DS of SPI-ODA and SPI-HFBAPP from H-NMR results are 41.6% and 42.3%, respectively. Stereo-hindrance effect leads to the DS being lower than that of the feed molar ratio.

(5) $$\;DS_{ODA}\;=\;\frac{\frac{A_{a}}{18}}{\frac{A_{b,c,d,e,f}\;-\;\frac{A_{a}}{18}\;\times \;10}{12}\;+\;\frac{A_{a}}{18}}\;$$

(6) $$\;DS_{HFBAPP}\;=\;\frac{\frac{A_{a}}{18}}{\frac{A_{b,c,d,e,f}\;-\;\frac{A_{a}}{18}\;\times \;10}{20}\;+\;\frac{A_{a}}{18}}\;$$

where *A_a_* and *A_b,c,d,e,f_* are the integral of H(a) and H(b,c,d,e,f), respectively.

### 3.2. Physicochemical Properties of SPI Membranes

**Scheme 2:**
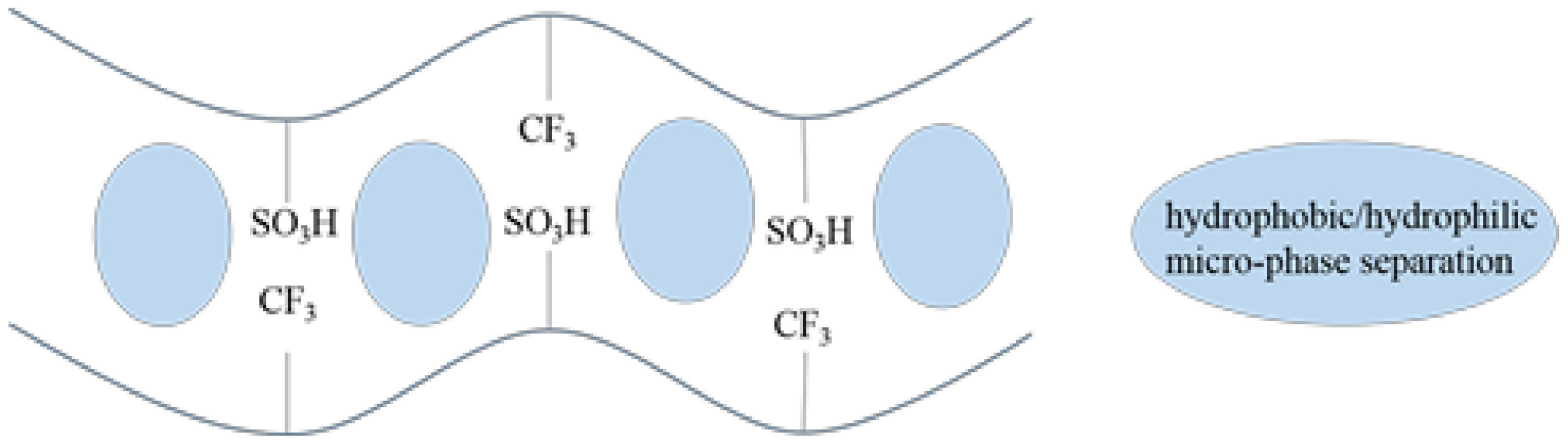
Micro-phase separation in SPI-HFBAPP membrane.

Some important parameters that could affect the cell performance of those membranes, such as WU, IEC, area resistance, and vanadium permeation were characterized and the results were summarized in Table 1. As can be seen, the WU of SPI-ODA membrane is just a little higher than that of N115 membrane, while the WU of SPI-HFBAPP membrane is much lower than that of N115 membrane because of the introduction of hydrophobic fluorine group –CF_3_. The IEC value of N115 membrane is inferior to SPI membranes, while the IEC is determined by the capacity of free ion exchange group in the membranes, therefore, SPI membranes could provide more ionic exchange groups during the process of proton migration. As a result, the area resistance of SPI membranes is lower than that of N115 membrane. Generally speaking, the increasing of WU and IEC would result in the development of proton conductivity. However, the WU and IEC of SPI-HFBAPP membrane are both lower than those SPI-ODA membrane; therefore, the proton conductivity of SPI-HFBAPP membrane is superior to SPI-ODA membrane. This may be due to fact that the fluorocarbon group and sulfonic functional group lead to the formation of hydrophobic/hydrophilic micro-phase separation in SPI-HFBAPP membrane, just as Scheme 2 shown, hence the protons are easier to transport in SPI-HFBAPP membrane. 

**Figure 3:**
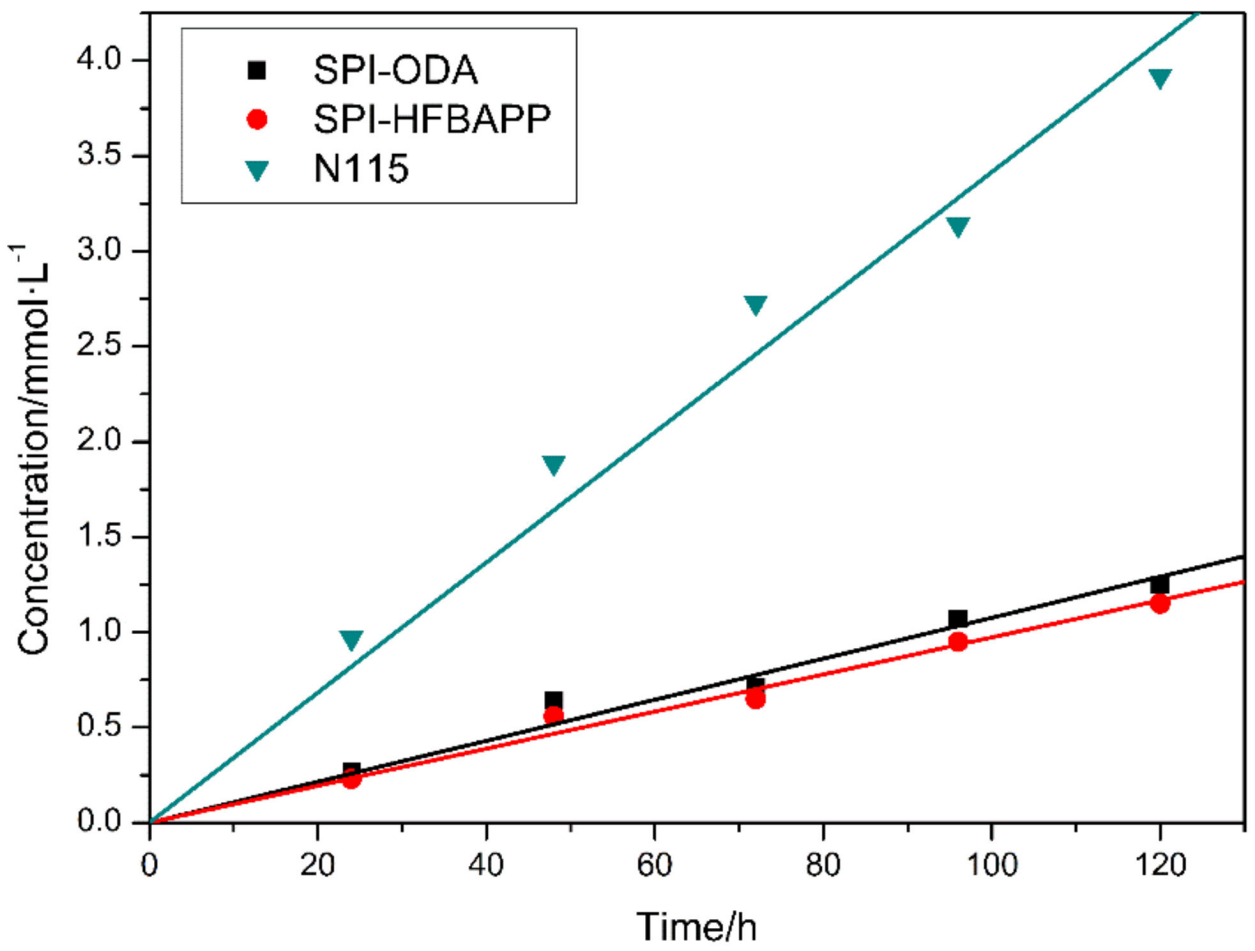
The vanadium ion concentration changes in MgSO_4_ solution across SPI and N115 membranes.

Vanadium permeation is an important parameter that represents the speed of vanadium traverse the membrane, and the crossover of vanadium will lead to the contamination of positive and negative electrolytes. Thus, the vanadium permeation would directly influence the CE and self-discharging time of the VFB. The change of vanadium ion concentration along with time is displayed in Figure 3. From the curves, the change of vanadium ion concentration for N115 membrane was obviously larger than that of SPI-ODA membrane and SPI-HFBAPP membrane. The corresponding vanadium ion permeation is listed in Table 1, the vanadium ion permeation of SPI-HFBAPP membrane is 0.97 × 10^−7^ cm^2^·min^−1^, only about one-tenth of N115 membrane. So it could be predicted that the CE and self-discharging time of SPI-HFBAPP membrane will significantly exceed N115 membrane, which is attributed to the fact that both of N115 and SPI-HFBAPP membranes are containing hydrophobic fluorocarbon and hydrophilic sulfonic groups, so they are have similar micro-phase separation caused by hydrophobic/hydrophilic groups. According to Liu and Jia [44,45], the micro-phase separation plays a great important role in ionic permeation, the hydrophilic sulfonic group in N115 membrane is longer than that of SPI-HFBAPP membrane, so the micro-phase separation degree of N115 membrane is larger, ultimately, both proton conductivity and vanadium ion permeation of N115 membrane are higher than those of SPI-HFBAPP membrane. Because there is no hydrophobic side chain for SPI-ODA membrane, there is no micro-phase separation in SPI-ODA membrane. Hence, the proton conductivity and vanadium permeability of SPI-ODA membrane is also lower than that of N115 membrane.

In most cases, proton conductivity and vanadium ion permeation are restricted to each other; increasing proton conductivity usually aggravates the vanadium permeability. The increasing of proton conductivity is better for cell performance, while the aggravation of vanadium permeability harms cell performance, so the optimal balance between proton conductivity and vanadium permeability is really important. Proton selectivity is often used to evaluate the balance between proton conductivity and vanadium permeability, and it is the ratio of proton conductivity to vanadium permeability. The proton selectivity of N115 membrane and SPI membranes are presented in Table 1, the proton selectivity of SPI-HFBAPP membrane is the highest, about eight times higher than that of N115 membrane. This result signifies that the dimension of micro-phase separation in SPI-HFBAPP membrane is more suitable to prepare IEM. Although there is no micro-phase separation in SPI-ODA, its proton selectivity was also higher than N115 due to the excellent vanadium blocking. 

**Figure 4:**
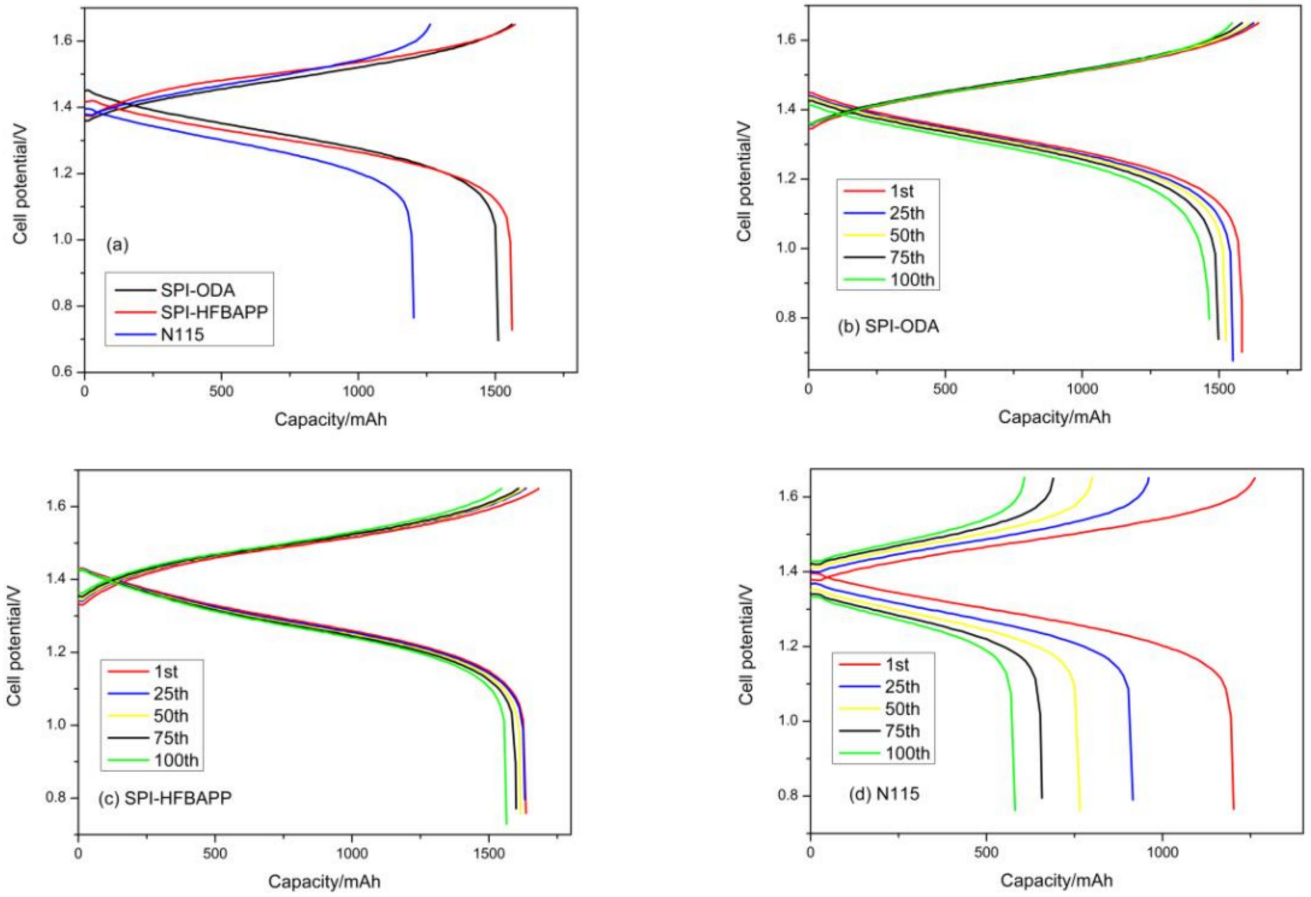
Charge–discharge cures of the VRFB with SPI membranes and N115 at current density of 100 mA·cm^−2^; (**a**) is the capacity of the first cycle while (**b**), (**c**), and (**d**) are the capacities of different cycles.

The typical charge–discharge curves of the VFB single cell assembled with SPI membranes and N115 membrane are exhibited in Figure 4. The cell capacity of VFB is mainly affected by the area assistance and vanadium ion permeation. The VFB single cell assembled with SPI-HFBAPP membrane shows the longest charge–discharge time and highest capacity at first cycle due to the low area resistance. While N115 membrane has the highest area resistance, so its charging capacity and discharging capacity are both the lowest at the first cycle. The vanadium permeability of N115 membrane is the highest; therefore, the capacity loss of N115 membrane is much more severe than those of SPI-ODA membrane and SPI-HFBAPP membrane. The charging capacity of N115 membrane is decreased from 1205 m Ah to 580 m Ah after 100 cycles, while for SPI-HFBAPP membrane it is only decreased from 1643 m Ah to 1557 m Ah after 100 cycles. This means that in practical application the replacement of electrolyte is less frequent for VFB assembled with SPI-HFBAPP membrane.

**Figure 5:**
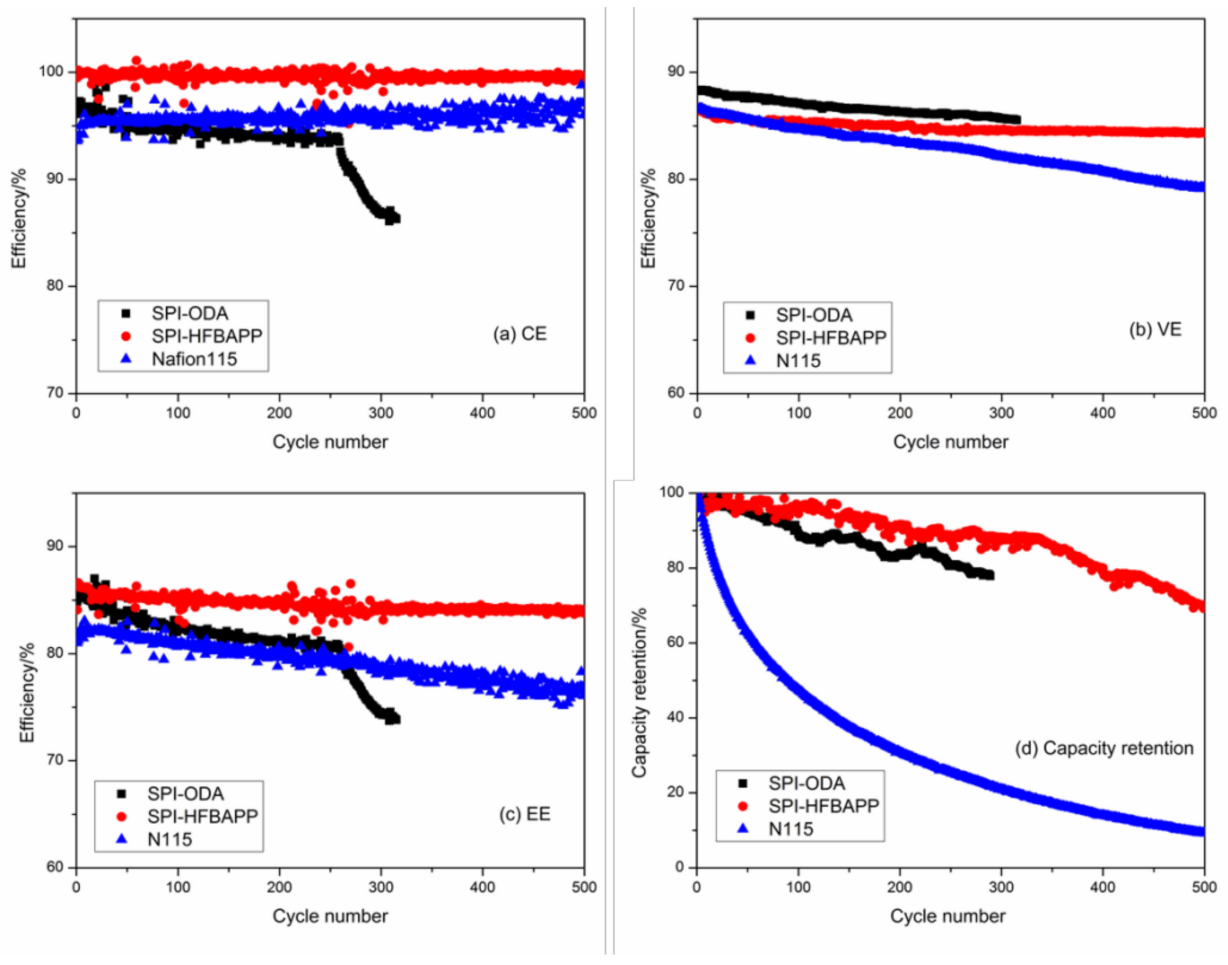
Cycling stability of the VRFB with SPI membranes and N115 at current density of 100 mA·cm^−2^. (**a**) CE; (**b**) VE; (**c**) EE; (**d**) Capacity retention.

The efficiency of the VFB single cell assembled with SPI membranes and N115 membrane under the current density of 100 mA·cm^−2^ are listed in Table 2 and Figure 5. When considering that the SPI-ODA membrane is broken after 250 cycles as shown in Figure 5, the values of all membranes in Table 2 are the average of 250 cycles. The CE of SPI-HFBAPP membrane could be as high as 99.5%, obviously higher than N115 membrane (CE = 95.5%). This result is consistent with the vanadium permeability; excellent vanadium resistance of SPI-HFBAPP membrane reduces the capacity loss during cycling, leading to high CE. Meanwhile, the capacity retention of SPI-HFBAPP membrane is evidently superior to N115 membrane after 500 cycles. While for the SPI-ODA membrane, the CE is 94.2%, lower than that of N115 membrane though the vanadium permeability of SPI-ODA membrane is less than N115. The poor oxidative resistance of SPI-ODA membrane made it gradually damaged during the cycling, so the CE of SPI-ODA membrane gradually decreased during the cycling. Just as Figure 5 shows, the CE of SPI-ODA membrane is higher than N115 membrane at the very beginning. As the cycling increases, the CE of SPI-ODA membrane gradually reduces while the CE of N115 membrane remains constant; after 50 cycles, the CE of N115 membrane exceeds SPI-ODA membrane. Ultimately, the average CE of N115 membrane is about 1.3% higher than that of SPI-ODA membrane. The VE of all membranes are comparatively high, the VE of SPI-HFBAPP membrane and SPI-ODA membrane are 85.3% and 86.7% respectively at current density of 100 mA·cm^−2^, both higher than N115 membrane (VE = 84.5%). As discussed above, the area resistance of SPI-HFBAPP membrane and SPI-ODA membrane are both lower than that of N115 membrane, thus the VE of SPI membranes are better. EE is a compressive parameter to evaluate the performance of VFB. Due to the fact that the VE of all membranes are similar, thus, the advantage of CE of SPI-HFBAPP is more significant, the EE of SPI-HFBAPP membrane is the uppermost, and could be reached 85.0%. These results demonstrate that SPI is a kind of suitable material for preparing IEM for VFB application and the introduction of fluorocarbon group could further improve the performance of the membrane.

**Figure 6:**
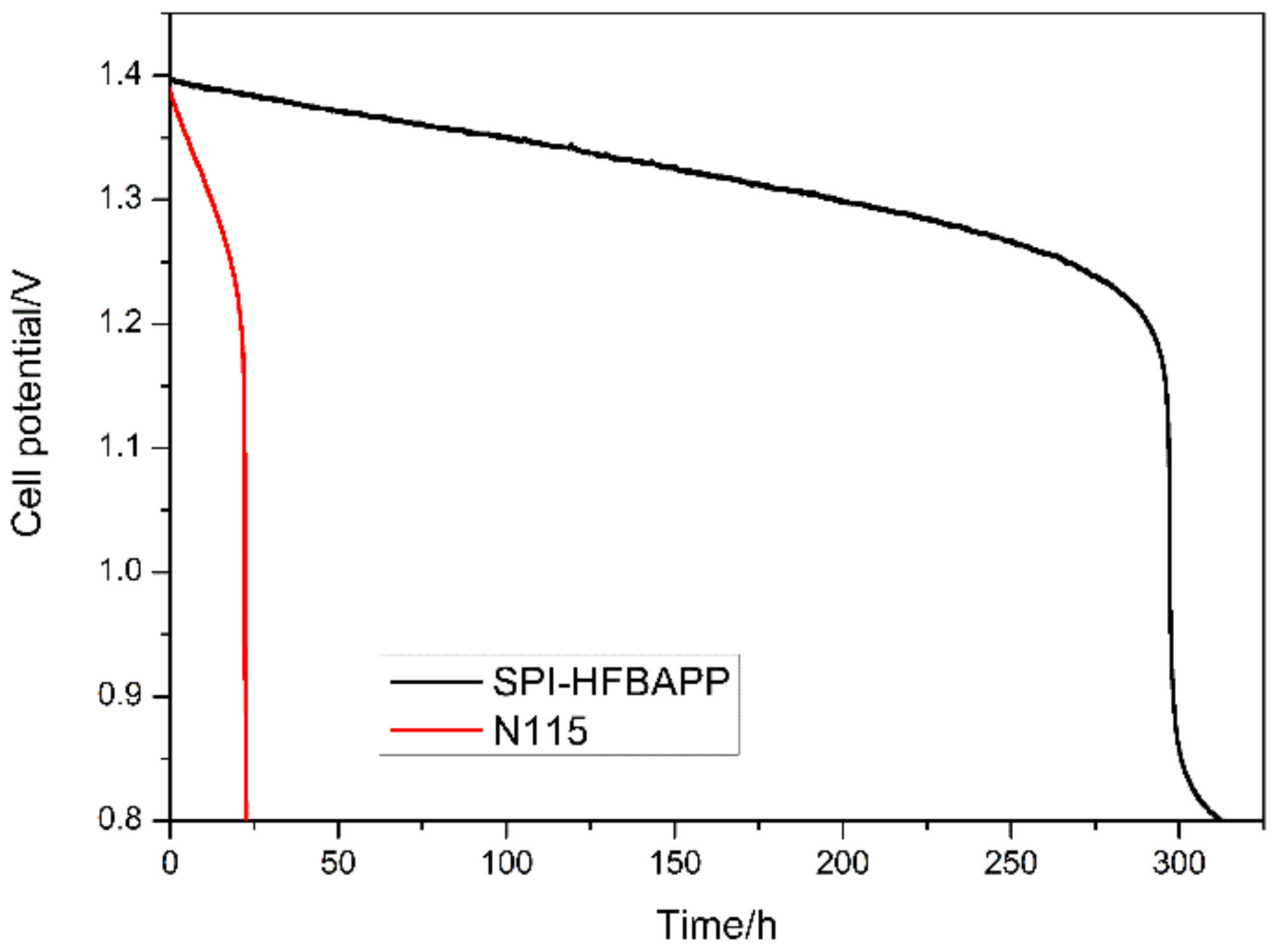
Self-discharge of the VRFB assembled with N115 and SPI-HFBAPP membranes.

The self-discharge of the battery is caused by vanadium ion contamination. It is usually evaluated by open circuit voltage (OCV). The cells were charged for 50% state at current density of 50 mA·cm^−2^, then self-discharge tests were conducted and finished when the OCV was dropped to 0.8 V. As Figure 6 shown, the self-discharge time of SPI-HFBAPP membrane is longer than 300 h, while for N115 membrane it is only about 22 h. The result further confirms that the vanadium restraint of SPI-HFBAPP membrane is excellent.

### 3.3. Stability of SPI Membranes

**Figure 7:**
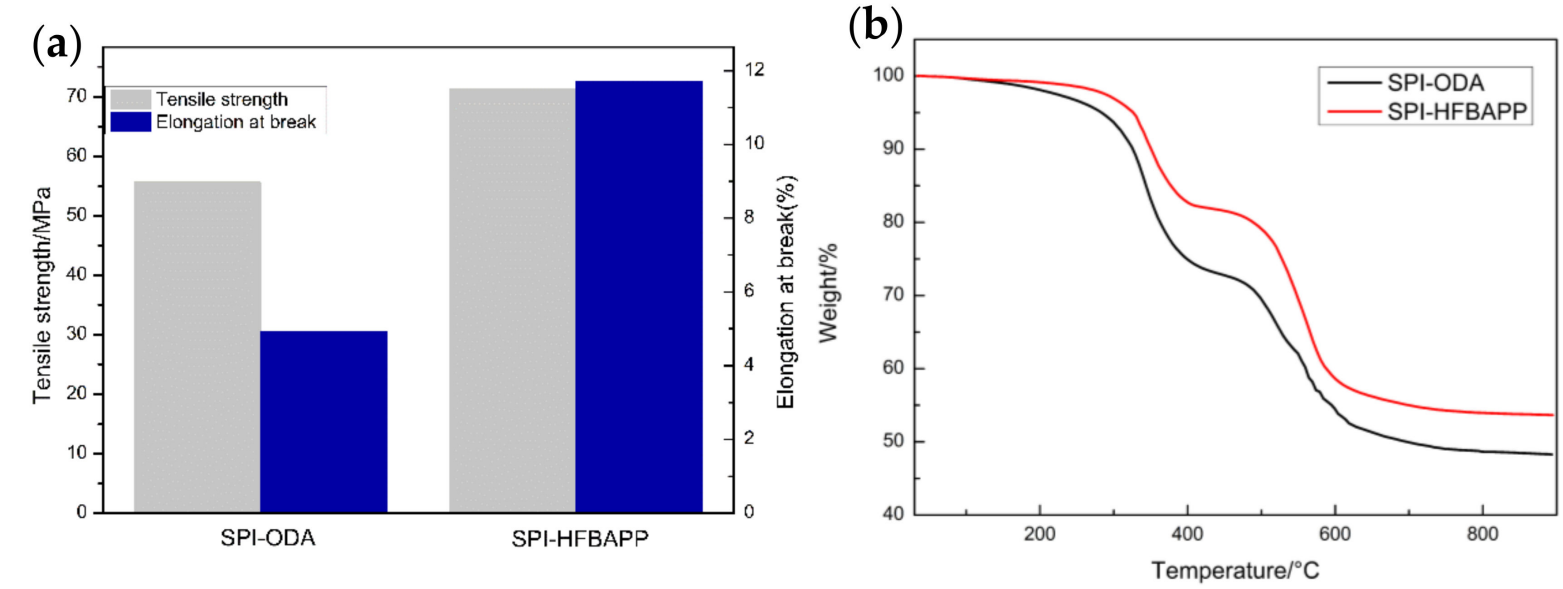
Mechanical and thermal stability of SPI membranes. (**a**) mechanical stability; (**b**) thermal stability.

The electrolytes of VFB are strongly acidic and oxidative. Hence, the stability of IEM is crucial for the application. The mechanical strength, thermal stability, and oxidative stability of SPI membranes are characterized. The mechanical strength of the SPI membranes plays an important role on its stability. The mechanical properties of SPI membranes are exhibited in Figure 7a. The rigid aromatic backbone could improve the tensile strength, so the tensile strength of SPI-ODA and SPI-HFBAPP membranes are well above 55 MPa. In addition, the fluorocarbon groups in HFBAPP lead to the increase of hydrophobicity and the reduction of WU (listed in Table 1). The low water uptake would result in high tensile strength. SPI-HFBAPP membrane has higher tensile strength due to low water uptake [46]. The SPI-HFBAPP membrane has higher elongation at break than SPI-ODA membrane because of its aliphatic main chain and ionic crosslinking. All of them would lead to the increase of elongation at break [47]. The introduction of fluorocarbon groups could improve the mechanical properties of SPI membrane.

The thermal stability of SPI membranes are displayed in Figure 7b. The initial thermal decomposition temperatures of both membranes are higher than 200 °C. The actual application temperature of VFB is 20–50 °C. The thermal stability of SPI membranes can meet the actual application.

**Figure 8:**
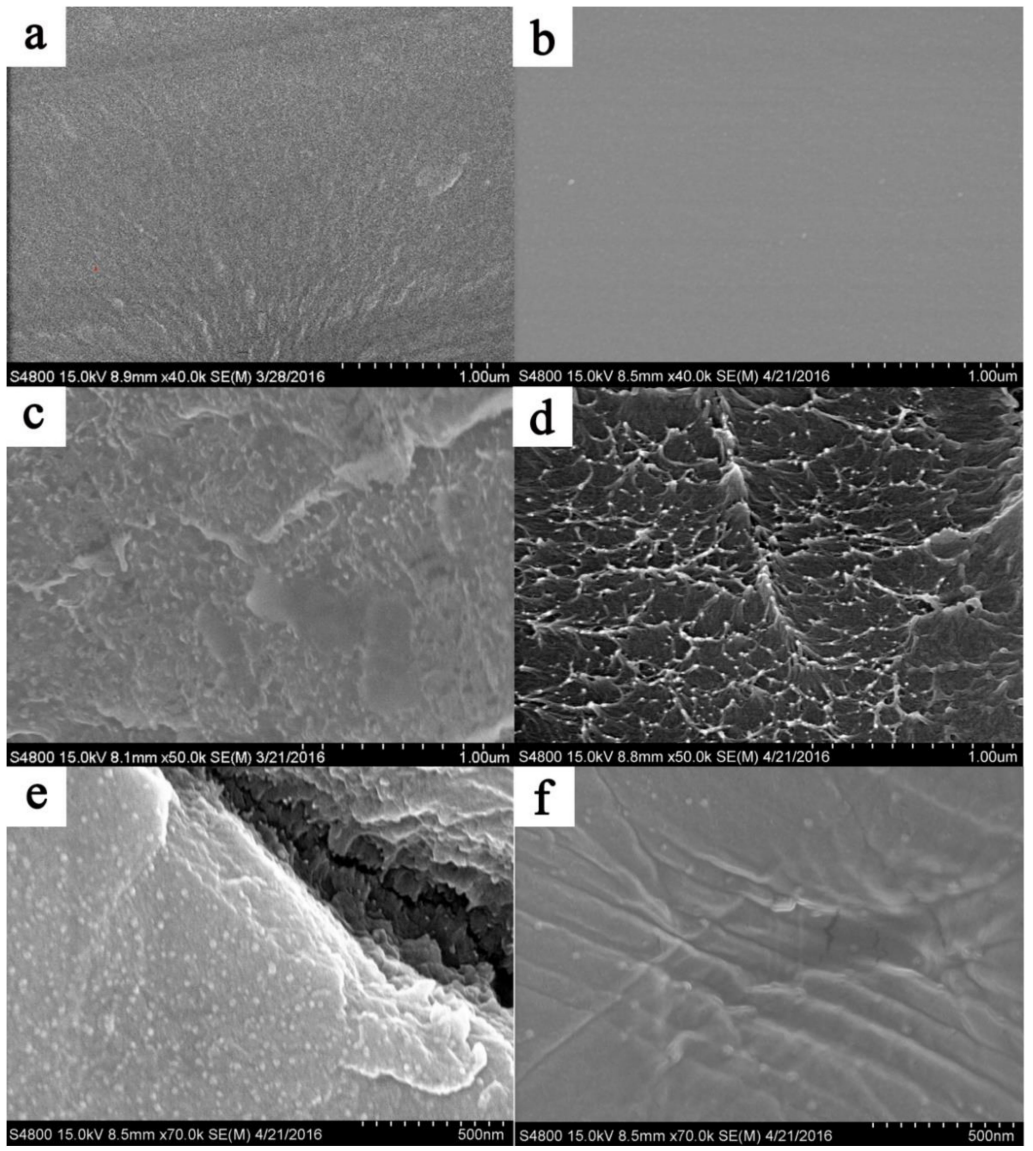
SEM micrographs of SPI membranes: (**a**) surface and (**b**) cross-section images of pristine SPI-ODA membrane; (**c**) surface and (**d**) cross-section images of pristine SPI-HFBAPP membrane; surface images of (**e**) SPI-ODA and (**f**) SPI-HFBAPP after cell cycling.

**Figure 9:**
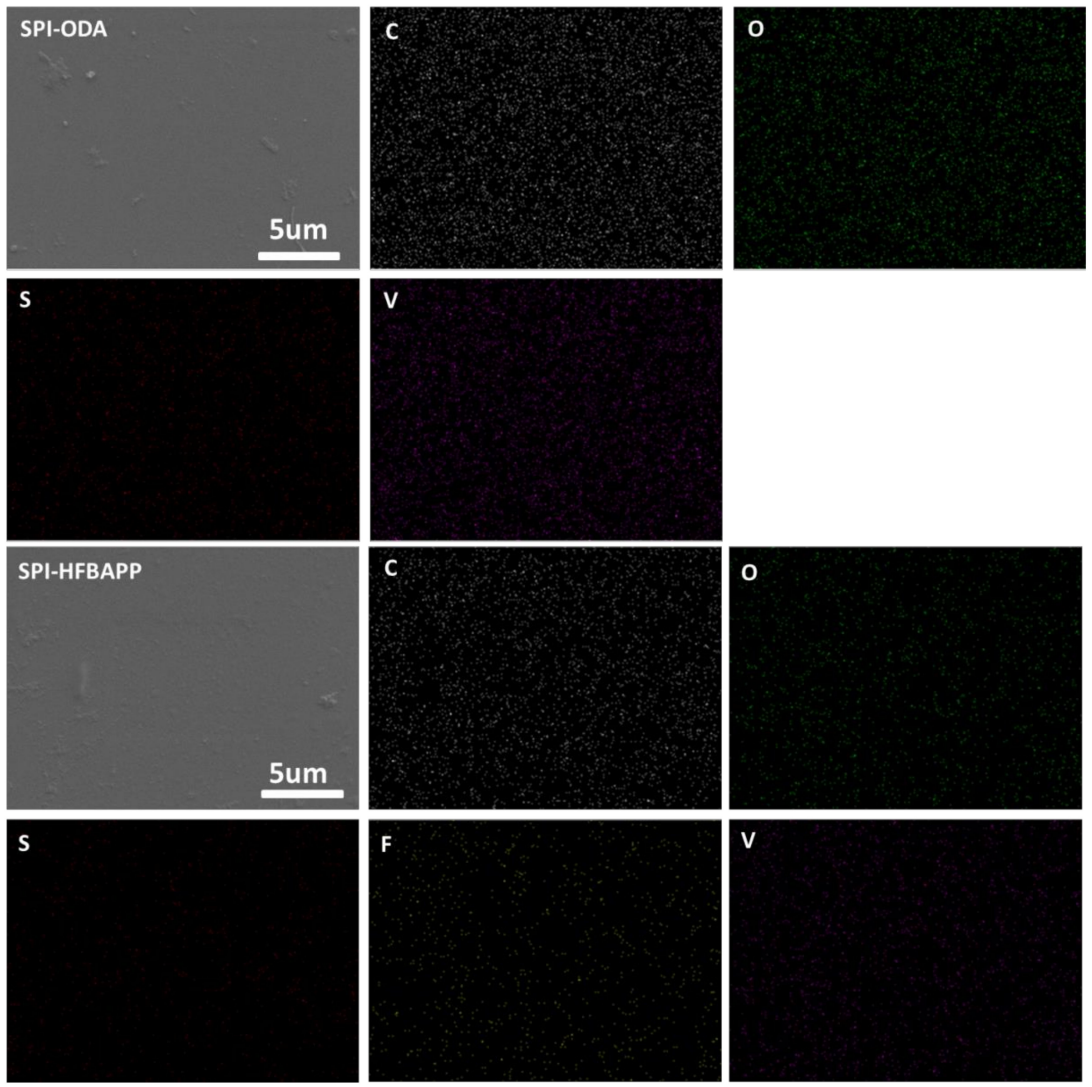
EDX images of SPI membranes after cell cycling. **C**: carbon; **O**: oxygen; **S**: sulfur; **V**: vanadium; **F**: flourin.

The oxidative stability of membranes is indirectly characterized by SEM images. As can be seen from Figure 8, the surface morphology of pristine SPI membranes is homogeneous, smooth, and dense, with no pores and cracks. There is no obvious difference between the surface morphology of SPI-ODA membrane and SPI-HFBAPP membrane. The cross-section of SPI-ODA membrane is homogeneous and dense, while there are some bumps on the cross-section of SPI-HFBAPP membrane. There are sharp cracks on the surface of SPI-ODA membrane after 250 cycles at current density of 100 mA·cm^−2^, while there are only minor cracks on the surface of SPI-HFBAPP membrane after 500 cycles at current density of 100 mA·cm^−2^. The introduction of fluoride groups effectively improved the oxidative resistance and hydrolysis resistance of the membrane. Thus, the water-soluble vanadium was harder to absorb on SPI-HFBAPP membrane. As shown in Figure 9, more vanadium ions absorbed on SPI-ODA membrane the SPI-HFBAPP membrane after cell cycles. The oxidizing pentavalent vanadium could do damage to the ether bonds in SPI. Therefore, the oxidative resistance of SPI-HFBAPP membrane was superior to SPI-ODA membrane and more adapted to the long running time. Even though the SPI-ODA membrane was damaged due to the oxidative environment of electrolyte and some vanadium ions appeared at the surface of the membrane, all elements were distributed uniformly as shown in Figure 9.

## 4. Conclusions

SPI-ODA and fluorinated SPI-HFBAPP were successfully synthesized and used to prepare membranes for VFB applications. The introduction of fluorocarbon groups leads to the formation of the hydrophobic/hydrophilic micro-phase separation and the ion channels, decreasing the area resistance of the membrane. In addition, the introduction of fluorocarbon groups enhanced the proton selectivity of the membrane. Hence, the VFB assembled with SPI-HFBAPP membranes displayed superior cell performance. The EE of VFB assembled with SPI-HFBAPP membrane was 84.8%. The introduction of fluorocarbon groups also improved the mechanical properties and oxidative stability of SPI-HFBAPP membrane, the CE and VE of VRFB assembled with SPI-HFBAPP membrane kept constant after cycling for 500 at current density of 100 mA·cm−2.The main chain of fluorinated SPI is rigid and the fluorocarbon groups are located in side chain, so the ion channels in the membranes were less than Nafion115. Thus, the properties of SPI-HFBAPP membrane, such as vanadium ion resistance and proton selectivity, were improved. The CE and VE of SPI-HFBAPP were both higher than N115. The advantage of vanadium resistance is especially noticeable. The self-discharge time of SPI-HFBAPP membrane could be as long as 300 h, about 13 times of N115.

## Figures and Tables

**Table 1:** Physicochemical property of N115 and SPI membranes

Membranes	Thickness (μm)	WU (%)	IEC (mmol·g^−1^)	Area Resistance (Ω·cm^2^)	Proton Conductivity (mS·cm^−1^)	VO^2+^ Permeation (·10^−7^ cm^2^·min^−1^)	Proton Selectivity (10^4^ S·min·cm^−3^)
N115	140	13.17	0.669	0.285	49.12	9.69	5.07
SPI-ODA	58	15.68	1.468	0.183	31.69	1.61	19.68
SPI-HFBAPP	58	6.66	1.232	0.154	37.66	0.97	38.82

**Table 2:** VRFB single cell performance of SPI and N115 membranes at the current density of 100 mA·cm^−2.^

Membranes	CE (%)	VE (%)	EE (%)
SPI-ODA	94.2	86.7	81.7
SPI-HFBAPP	99.7	85.3	85.0
N115	95.5	84.5	80.7
